# The prevalence of headache disorders in children and adolescents in Nepal: a schools-based cross-sectional study

**DOI:** 10.1186/s10194-025-02129-6

**Published:** 2025-10-16

**Authors:** Rajeev Ojha, Ragesh Karn, Bikram Prasad Gajurel, Reema Rajbhandari, Niraj Gautam, Bikash Deo, Aakarshan Timilsina, Anushka Adhikari, Ravi Raj Timasina, Gaurav Nepal, Tayyar Şaşmaz, Bengü Nehir Buğdaycı Yalçın, Amalie Berring-Uldum, Andreas Kattem Husøy, Derya Uludüz, Timothy J. Steiner

**Affiliations:** 1 Department of Neurology, Tribhuvan University Teaching Hospital, Kathmandu, Nepal; 2 Department of Medicine, Tribhuvan University Teaching Hospital, Kathmandu, Nepal; 3 Department of Medicine, Lamjung District Hospital, Besisahar, Nepal; 4 Department of Psychiatry, Gandaki Medical College, Pokhara, Nepal; 5 Department of Public Health, Mersin University School of Medicine, Mersin, Turkey; 6 Department of Pediatrics and Adolescent Medicine, Copenhagen University Hospital - Herlev and Gentofte, Herlev, Denmark; 7 Faculty of Health and Medical Sciences, University of Copenhagen, Copenhagen, Denmark; 8 Norwegian Centre for Headache Research (NorHead), Department of Neuromedicine and Movement Science, Norwegian University of Science and Technology, Trondheim, Norway; 9 Department of Neurology and Clinical Neurophysiology, St Olavs University Hospital, Trondheim, Norway; 10 Neurology Department, Cerrahpaşa School of Medicine, Istanbul University, Istanbul, Turkey; 11 Department of Neurology, University of Copenhagen, Copenhagen, Denmark; 12 Division of Brain Sciences, Imperial College London, London, UK

**Keywords:** Headache disorders, Migraine, Tension-type headache, Medication-overuse headache, Undifferentiated headache, Epidemiology, Children, Adolescents, Burden of disease, Schools-based survey, Nepal, South East Asia region, Global Campaign against Headache

## Abstract

**Background:**

Knowledge of headache among children (6–11 years) and adolescents (12–17 years) remains relatively sparse. This schools-based, cross-sectional survey in Nepal, in the series of similar studies conducted within the Global Campaign against Headache, was the first in South-East Asia Region. Our aims were to estimate the prevalence among these age groups of each of the headache disorders of public-health importance, and analyse their associations with demographic variables.

**Methods:**

The study followed the Global Campaign’s standardized protocol. In schools selected to be representative of the country, the child and adolescent versions of the structured Headache-Attributed Restriction, Disability, Social Handicap and Impaired Participation (HARDSHIP) questionnaire were completed by pupils in class under supervision. Headache diagnoses followed ICHD-3, with the exception of undifferentiated headache (UdH), which, initially, we defined conventionally as mild headache lasting < 1 h, then redefined as mild-to-moderate headache lasting < 1 h. The diagnosis of UdH took precedence over diagnoses of migraine or tension type headache (TTH). Enquiry timeframes were 1 year and 4 weeks, with questions about headache yesterday (HY) included to circumvent recall error.

**Results:**

Of 2,360 potential participants in nine schools, 2,352 completed the survey (1,040 children [44.2%]; 1,312 adolescents [55.8%]; participating proportion 99.7%). Males (54.0%) were slightly over-represented. The observed 1-year prevalence of any headache was 85.4% (males 83.5%; females 87.5%). Age- and gender-adjusted estimates were 82.4% for any headache, 39.9% for migraine, 30.3% for UdH (as redefined), 9.5% for TTH, 0.3% for probable medication-overuse headache (pMOH) and 2.0% for other headache on ≥ 15 days/month (H15+). Gender was not strongly associated with any headache type. UdH, TTH and other H15 + were more common among adolescents. Migraine was strongly and positively associated with altitude; the opposite was true for UdH. HY was reported by 20.7% of the sample (1-day prevalence), and by 24.3% of those with any headache (females [29.6%] more than males [19.5%]). The 24.3% was higher than predicted from headache frequency recalled over the preceding 1 week (17.6%) or 4 weeks (10.3%), indicating that participants, in recall, underestimated frequency.

**Conclusions:**

This study, complementing our adult study, showed that headache is highly prevalent also among children and adolescents in Nepal. As among adults, migraine was strongly and positively associated with altitude. The finding of less pMOH and other H15 + among these young people suggests opportunity to avert some of the headache-attributed burden among adults.

## Introduction

According to estimates from the Global Burden of Disease, Injuries and Risk Factors (GBD) study, headache disorders are among the top causes of heath loss in children and adolescents [1, 2]. These estimates are based on rather sparse data: globally, the prevalence and attributed burden of headache disorders in those aged < 18 years are very incompletely described. Specifically to address this knowledge gap, the Global Campaign against Headache commenced a programme of schools-based studies in countries around the world, completing studies in Benin [3], Ethiopia [4], Lithuania [5], Mongolia [6], Turkey [7] and Zambia [8] before the programme was interrupted by the covid-19 pandemic. This study in Nepal, conducted after schools reopened, is the first in the programme from South East Asia Region (SEAR). While no previous studies of any sort have addressed childhood and adolescent headaches in Nepal, a previous population-based survey of adults in this country revealed a very high burden of headache, and a strong association between migraine and altitude [9, 10].

Nepal’s population is young: almost 40% are under the age of 18 years [11]. Net enrolment rate in primary school (6–11 years) in 2022 was 99.0%, with > 90% transitioning to upper primary (12–14 years; net enrolment 91.7%), and > 75% to secondary school (15–18 years; net enrolment 76.0%) [12, 13]. Schools-based studies are therefore an effective and economical way of surveying these age groups, with the potential to reach all children and most adolescents.

The programme is focused on the most common headache types – and those of greatest public-health importance: migraine, tension-type headache (TTH) and headaches occurring on ≥ 15 days/month (H15+), including medication-overuse headache (MOH), along with undifferentiated headache (UdH), the short-lasting headaches believed to represent manifestations of migraine or TTH in the developing brain [7, 14]. Previous studies in other countries have found UdH to be prevalent in both children and adolescents [3–8].

The purposes of this study were to enhance understanding of the global headache burden in children and adolescents and to inform public-health and educational policies in Nepal. Here we report the prevalence of various headache types and their associations, while a subsequent paper will focus on attributed burden and consequences of these disorders.

## Methods

We followed the programme’s standardised protocol [14] in a cross-sectional survey conducted in schools across Nepal. Pupils filled out a structured questionnaire in class, under the supervision of school staff.

### Ethics and approvals

The protocol was approved by the Institutional Review Committee of Tribhuvan University Institute of Medicine: 169(6–11)F2 078/079. Necessary authorizations from academic and administrative authorities, and permissions from principals and class teachers, of each school were obtained.

In accordance with the ethics approval, all participants (pupils and teachers) were informed verbally of the nature and purpose of the study, and gave oral consent before enrolment. Continuing consents were evidenced by active completion of the questionnaires. In view of the non-interventional nature of the study, neither the Institutional Review Committee nor the academic and administrative authorities requested parental consent.

Data were recorded anonymously, and managed in accordance with local data protection regulations.

### Sampling and recruitment

This study was carried out from March to November 2022.

In accordance with published guidelines [14, 15], we adopted a cluster-sampling methodology and aimed for 2,100 participants (about 175 from each year of age).

**Fig. 1:**
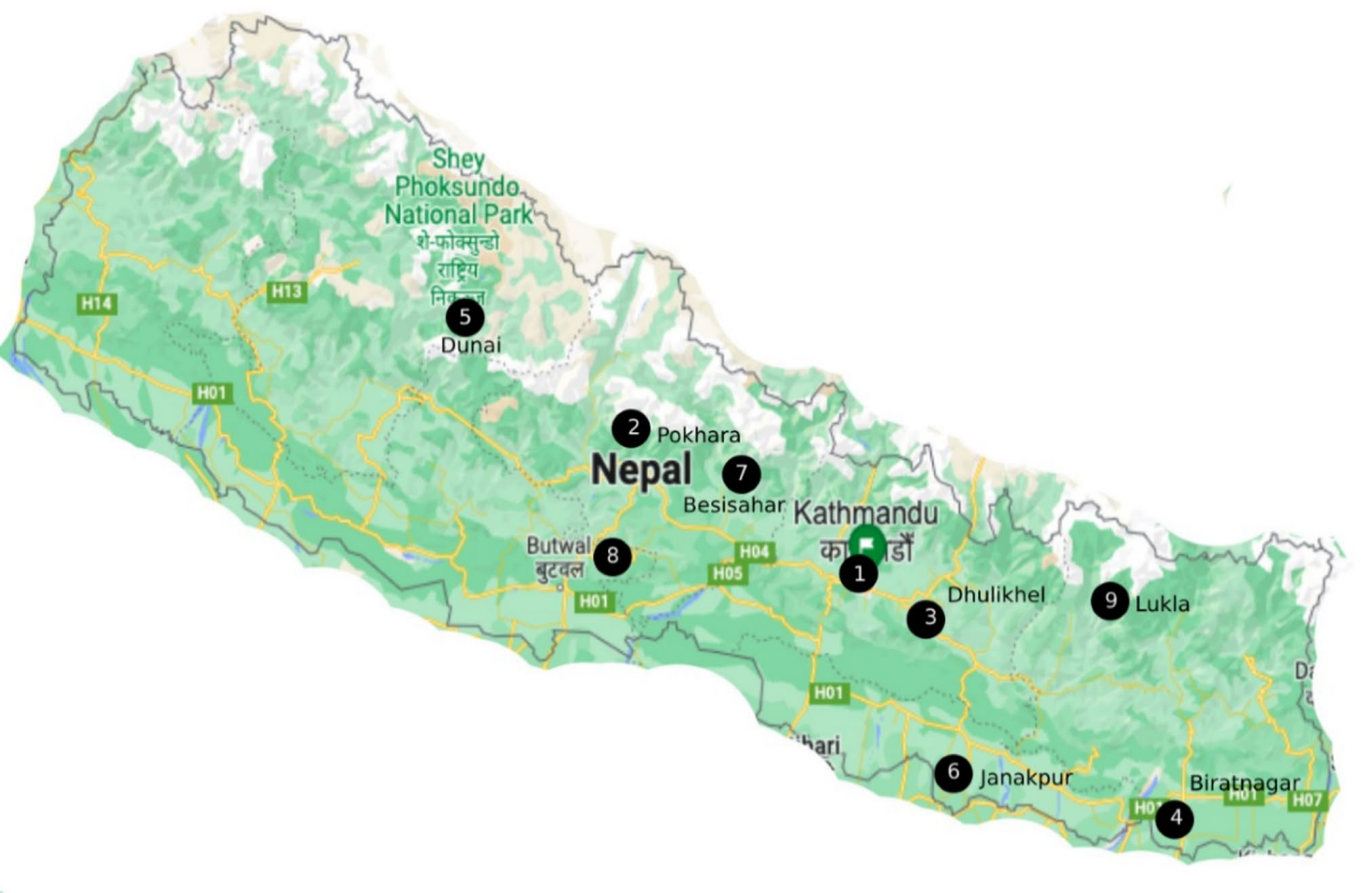
Distribution of selected schools in nine districts of Nepal

We included nine schools representing a mix of urban and rural populations across the socioeconomic range and from altitudes 70 to 2,860 m above sea level. They were situated in nine districts of Nepal, each in the administrative centres of six of Nepal’s seven provinces (Biratnagar and Lukla in province 1, Janakpur in province 2, Kathmandu and Dhulikhel in province 3, Pokhara and Besisahar in province 4, Butwal in province 5 and Dunai in province 6 [Fig. 1]). Province 7 in the far western region was not reachable, and administrative approvals from schools in the province were not forthcoming.

We rescheduled data collection in Lukla three times because of bad weather (the airport of Lukla is regarded as one of the most dangerous airports in the world, with flights allowed only in good visibility). In Besisahar, we rescheduled twice because of news of landslides affecting unpaved roads.

In each school included, pupils aged 6–11 years (children) or 12–17 years (adolescents) and present on the day of survey were eligible for inclusion and invited to participate. Those who declined or could not participate were counted as non-participants. Those absent from school on the survey day were not available for inclusion and therefore not counted as non-participants.

### Data collection

We used the child and adolescent versions of the Headache-Attributed Restriction, Disability, Social Handicap and Impaired Participation (HARDSHIP) questionnaire, each containing 45 questions [14]. These were translated into Nepali following the Global Campaign’s translation guidelines for lay documents [16], with a pilot study in 155 children and 119 adolescents to assess usability and comprehensibility in these age groups. The questionnaires began with demographic enquiry, followed by headache screening questions. For those who reported headache in the preceding year, diagnostic questions were posed based on ICHD-3 criteria [17], followed by enquiries into attributable burden (not reported here). The timeframes of these enquiries were the preceding 1 year and 4 weeks (28 days), but additional questions about headache on the preceding day (headache yesterday [HY]) were included to identify and circumvent recall error [15].

The questionnaires were distributed to the pupils in class, who completed them under supervision by the researchers and class teacher. Guidance and clarification were provided when needed.

Separate questionnaires, completed by the teachers, enquired into each school’s location (urban or rural) and altitude, and the estimated proportions of pupils coming from low-income homes and travelling for > 1 h each day.

### Headache diagnoses

Diagnoses were made by applying the HARDSHIP algorithm [7, 18] to the responses to the diagnostic question set. After identifying pupils reporting headache on ≥ 14 days in the previous 28 days (assumed to equate to ≥ 15 days in the previous 30 days [H15+]), we diagnosed those also reporting medication use on ≥ 14 days as probable MOH (pMOH); the others were not further diagnosed but categorised as “other H15+”. Among the remainder, we first identified UdH, initially as headache of mild intensity and usual duration of < 1 h (the conventional definition [7]), but subsequently as mild-to-moderate headache lasting < 1 h (see Results and Discussion). The diagnoses of pMOH, other H15 + and UdH therefore took precedence over diagnoses of definite migraine, definite TTH, probable migraine or probable TTH [7, 18], these derived by application of modified ICHD-3 criteria [17]. Definite and probable migraine were combined, as were definite and probable TTH, in subsequent analyses.

### Data management

Questionnaires were securely stored at the Department of Neurology, Tribhuvan University Teaching Hospital. Data were entered into SPSS software platform 25.0. To ensure accuracy, double data entry was performed, and discrepancies reconciled by cross-referencing to the original data.

### Analysis

The principal analysis was performed at University of Mersin, using the analytical methods adopted by all previous Global Campaign studies [3–8]. Additional statistical modelling to review, and revise, the diagnostic criteria for UdH was performed at NorHead.

We categorised schools by location (urban, semirural or rural) and, as a socio-economic indicator, by teachers’ estimates of proportions of pupils coming from low-income homes (< 25%, 25–49%, ≥ 50% [for simplicity we refer to these categories as “high-income”, “middle-income” and “low-income”]). We also characterised schools by proportions of pupils living at distances of > 1 h’s travel time.

In order to establish representativeness, we compared the sample with the population aged 6–17 in Nepal for demographic variables, using data from the World Bank [19].

For descriptive statistics, we used means and standard deviations (SDs) for continuous variables, and proportions (%) with 95% confidence intervals (CIs) for categorical variables.

We used conservative imputation for missing responses to the diagnostic questions: headache intensity was assumed to be moderate (this being neutral between migraine and TTH); nature of headache was assumed to be pressing when applying the algorithm for migraine (tending towards TTH), throbbing when applying the algorithm for TTH (tending towards unclassified); nausea and vomiting were assumed to be absent (since they were very likely to be reported if present).

We calculated observed prevalence of each headache type in the sample, and made population estimates by adjusting for gender and age, again using data from the World Bank [19]. To examine relationships between headache types and gender, age, altitude of dwelling and urban or rural habitation, we performed both bivariate analyses, reporting odds ratios (ORs), and binary logistic regression, reporting adjusted ORs (aORs).

We calculated the 1-day prevalence of any headache as the proportion of the entire sample reporting HY. We also calculated the proportions reporting HY of those with headache (any, and of each type) in the preceding year, along with the proportions predicted to have HY from the responses to two questions: “On how many days in the last week did you have headache?” and “On how many days in the past four weeks did you have headache?” The analyses by headache type assumed that HY was of the type described in earlier questioning.

The significance level for all analyses was set at *p* < 0.05.

## Results

The pilot study (in 155 children and 119 adolescents) assessing usability and comprehensibility of the questionnaires found no need for modifications.

A total of 2,360 pupils (children 1,045; adolescents 1,315) were present on the survey days, available for inclusion. Of these, eight (five children, three adolescents) were non-participants, either providing incomplete responses or being unable to follow repeated instructions. The total sample was *N* = 2,352 (children 1,040 [44.2%]; adolescents 1,312 [55.8%]), with an overall participating proportion of 99.7%.

The sample had a preponderance of males (1,269; 54.0%) in comparison with the population aged 6–17 years (50.9% [19]; *X*^*2*^(1, 2352) = 4.4, *p* = 0.04). Mean age was 11.8 years (SD = 2.7), very similar to but nonetheless significantly higher than the mean age of this population (11.5 years [19]; t-test (df = 2351) = 5.4; *p* < 0.001).

Table 1 shows information about the schools provided by the teachers. Urban schools were attended by rather more than half of pupils (55.4%), with fewer than 25% at these schools travelling for > 1 h to reach them. Schools located at or above 1,000 m above sea level were attended by a similar proportion (55.5%; 24.5% >2,000 m). Two thirds (68.5%) of pupils attended high-income schools (as defined: see Methods); none were judged to come from low-income homes (Table 1).

**Table 1:** School variables (from teachers’ questionnaires)

Variable of interest	Schools (*N* = 9) *n*	Pupils (*N* = 2,352) *n* (%)
School location		
urban semirural rural	5 2 2	1,302 (55.4) 474 (20.2) 576 (24.4)
School altitude (metres above sea level)		
< 1,000 1,000–2,000 > 2,000	4 3 2	1,048 (44.6) 728 (31.0) 576 (24.5)
Income category according to estimated proportion of pupils from low-income homes		
< 25% (“high-income” school) 25–50% (“middle-income” school) ≥ 50% (“low-income” school)	6 3 0	1610 (68.5) 742 (31.5) 0
Estimated proportion of pupils travelling > 1 h		
< 25% 25–50% ≥ 50%	5 4 0	1,302 (55.4) 1,050 (44.6) 0

### Headache prevalence

Headache ever was reported by 2,138 participants, a lifetime prevalence of any headache of 90.9%. Of these, 2,008 (85.4% [95% CI: 83.9–86.8%]) reported headache in the preceding year, rather fewer males (83.5% [81.4–85.5%]) than females (87.5% [85.4–89.4%]) (Table 2).

**Table 2:** Observed 1-year prevalence by headache type and gender, according to conventional criteria for undifferentiated headache (mild headache lasting < 1 h)

Headache type	Overall	Male	Female
	% [95% confidence interval]		
Any headache	85.4 [83.9–86.8]	83.5 [81.4–85.5]	87.5 [85.4–89.4]
Migraine	58.4 [56.4–60.4]	57.9 [55.1–60.7]	58.9 [55.9–61.9]
definite	14.8 [13.4–16.3]	14.7 [12.9–16.8]	14.9 [12.8–17.2]
probable	43.6 [41.6–45.6]	43.2 [40.4–46.0]	44.0 [41.1–47.1]
TTH	16.5 [15.0–18.0]	16.2 [14.2–18.3]	16.8 [14.6–19.2]
definite	6.4 [5.4–7.5]	6.9 [5.6–8.4]	5.8 [4.5–7.4]
probable	10.1 [8.9–11.4]	9.3 [7.8–11.1]	11.0 [9.2–13.0]
pMOH	0.3 [0.1–0.6]	0.3 [0.1–0.8]	0.3 [0.1–0.8]
Other H15+	2.0 [1.5–2.7]	1.6 [1.0-2.4]	2.5 [1.6–3.6]
UdH	4.8 [3.9–5.7]	4.9 [3.8–6.2]	4.6 [3.4-6.0]
Unclassified	3.5 [2.8–4.3]	2.7 [1.9–3.7]	4.4 [3.3–5.8]

*TTH* tension type headache, *pMOH* probable medication overuse headache, *H15+* headache on ≥ 15 days/month, *UdH* undifferentiated headache

Responses to the headache diagnostic questions were complete except for headache intensity (missing in one), throbbing/pressing (missing in two), nausea (missing in one), and vomiting (missing in one). For these, we used imputation (see Methods).

The conventional diagnostic criteria for UdH (mild headache lasting < 1 h [5]) were fulfilled by only 4.8% (3.9–5.7%) (Table 2); overall, only 7.8% reported headache of mild intensity (Table 3). With these criteria applied, 58.4% (56.4–60.4%) fulfilled those for migraine (definite 14.8% [13.4–16.3]; probable 43.6% [41.6–45.6]) (Table 2), a proportion driven by reported intensity (92.2% moderate or severe), worsening on exercise (61.1%) and nausea (59.0%), with phonophobia a near-universal experience (94.6%) during headache episodes of any type (Table 3).

**Table 3:** Proportions of participants with headache reporting specific headache characteristics

Characteristic	*n* (%)
Location	
unilateral	369 (18.4)
bilateral	1,639 (81.6)
Pain characteristic	
pulsating	828 (41.2)
pressing	1,178 (58.7)
Intensity	
mild	156 (7.8)
moderate	1,285 (64.0)
severe	566 (28.2)
Worse on exercise	1,227 (61.1)
Avoid exercise	945 (47.1)
Nausea	1,185 (59.0)
Vomiting	864 (43.0)
Photophobia	1,034 (51.5)
Phonophobia	1,900 (94.6)

Our modified criteria for UdH (mild-to-moderate headache lasting < 1 h [see Methods]) were fulfilled by 32.1% (30.2–34.0%), again rather fewer males (30.3% [27.8–32.8%]) than females (34.3% [31.5–37.1%]) (Table 4). With these modified criteria applied, migraine remained the most prevalent headache type (observed prevalence 39.8% [37.8–41.8%]; definite remaining at 14.8% [13.4–16.3]; probable reduced substantially to 25.1% [23.4–27.0]), with no gender difference (males 39.6% [36.9–42.3%]; females 40.1% [37.2–43.0]). Still contributing to these high estimates were the large proportions reporting nausea (59%) and/or vomiting (43.0%), and also, since phonophobia was almost universal (94.6%), those reporting photophobia (51.5%) (Table 3). Observed prevalence of TTH was also reduced, from 16.5% (15.0–18.0) (Table 2) to 10.5% (9.3–11.7%; definite 6.4% [5.4–7.5], probable 4.1% [3.3-5.0]). Observed prevalence of pMOH remained at 0.3% (0.1–0.5%), and of other H15 + at 2.0% (1.4–2.6) (Table 4). Adjustments for age and gender slightly reduced prevalence estimates for all headache (83.9%), migraine (39.5%), TTH (10.0%) and UdH (31.7%) (Table 4). With the modified criteria for UdH, only 0.6% of headaches remained unclassified.

**Table 4:** One-year prevalence by headache type and demographic variable according to modified criteria for undifferentiated headache (mild-to-moderate headache lasting < 1 h)

Variable	Any headache	Migraine	TTH	pMOH	Other H15+	UdH
	% [95% confidence interval]					
**Observed**						
Overall (*N* = 2,352)	85.4 [84.0-86.8]	39.8 [37.8–41.8]	10.5 [9.3–11.7]	0.3 [0.1–0.5]	2.0 [1.4–2.6]	32.1 [30.2–34.0]
Gender						
male (*n* = 1,269) female (*n* = 1,083)	83.5 [81.5–85.5] 87.5 [85.5–89.5]	39.6 [36.9–42.3] 40.1 [37.2–43.0]	11.3 [9.6–13.0] 9.6 [7.9–11.3]	0.3 [0.0-0.6] 0.3 [0.1–0.6]	1.6 [0.9–2.3] 2.5 [1.6–3.4]	30.3 [27.8–32.8] 34.3 [31.5–37.1]
Age						
6–11 (*n* = 1,040) 12–17 (*n* = 1,312)	77.3 [74.8–79.9] 91.8 [90.3–93.3]	37.8 [34.9–40.8] 41.5 [38.8–44.2]	7.4 [5.8-9.0] 13.0 [11.2–14.8]	0.5 [0.1–0.9] 0.2 [0.0-0.4]	1.3 [0.6-2.0] 2.6 [1.7–3.5]	29.7 [26.9–32.5] 34.1 [31.5–36.7]
School altitude (metres above sea level)						
< 1,000 (*n* = 1,048) 1,001–2,000 (*n* = 728) > 2,000 (*n* = 576)	84.3 [82.0-86.5] 84.3 [81.7–87.0] 88.7 [86.1–91.3]	31.2 [28.4–34.0] 45.7 [42.1–49.4] 48.6 [44.5–52.7]	11.0 [9.1–12.9] 10.6 [8.3–12.8] 9.4 [7.0-11.8]	0.1 [0-0.3] 0.5 [0.1–1.1] 0.3 [0-0.8]	1.1 [0.5–1.8] 4.3 [2.8–5.7] 0.7 [0.0-1.4]	40.3 [37.3–43.2] 22.8 [19.8–25.9] 29.0 [25.3–32.7]
School location						
urban (*n* = 1,302) semirural (*n* = 474) rural (*n* = 576)	83.2 [81.2–85.2] 87.3 [84.3–90.3] 88.7 [86.1–91.2]	32.3 [29.8–34.8] 50.0 [45.5–54.5] 48.4 [44.3–52.5]	11.4 [9.7–13.1] 9.3 [6.7–11.9] 9.5 [7.1–11.9]	0.2 [0.0-0.4] 0.4 [0.0–1.0] 0.3 [0.0-0.8]	1.4 [0.8-2.0] 5.3 [3.3–3.7] 0.7 [0.0-1.4]	37.3 [34.7–39.9] 21.7 [18.0-25.4 29.0 [25.3–32.7]
**Age- and gender-adjusted estimates**						
Overall	83.9 [82.4–85.4]	39.5 [37.5–41.5]	10.0 [8.8–11.2]	0.3 [0.1–0.5]	1.9 [1.4–2.5]	31.7 [29.8–33.6]

*TTH* tension type headache, *pMOH* probable medication overuse headache, *H15+* headache on ≥ 15 days/month, *UdH* undifferentiated headache

### Demographic associations

These, with the modified criteria for UdH applied, are shown in Tables 5 and 6.

**Table 5:** Unadjusted analyses of associations (bivariate analysis) between headache types and demographic variables according to modified criteria for undifferentiated headache (mild-to-moderate headache lasting < 1 h)

Variable	Migraine	TTH	pMOH	Other H15+		UdH
	Odds ratio [95% confidence interval]; *p*					
Gender						
male (*n* = 1,269) female (*n* = 1,083)	reference 1.0 [0.9–1.2] *p* = 0.86	reference 0.8 [0.6–1.1] *p* = 0.21	reference 0.9 [0.2-4.0] *p* = 0.87	reference 1.6 [0.9–2.9] *p* = 0.12		reference 1.2 [1.0-1.4] *p* = 0.05
Age (years)						
6–11 (*n* = 1,040) 12–17 (*n* = 1,312)	reference 1.2 [1.0-1.4] *p* = 0.08	reference 1.9 [1.4–2.5] ***p*** **< 0.001**	reference 0.3 [0.04–1.5] *p* = 0.17	reference 2.1 [1.1–4.2] ***p*** **= 0.02**		reference 1.2 [1.0-1.5] ***p*** **= 0.02**
School altitude (metres above sea level)						
< 1,000 (*n* = 1,048) 1,001–2,000 (*n* = 728) > 2,000 (*n* = 576)	reference 1.9 [1.5–2.3] ***p*** **< 0.001** 2.1 [1.7–2.6] ***p*** **< 0.001**	reference 1.0 [0.7–1.3] *p* = 0.79 0.8 [0.6–1.2] *p* = 0.31	reference 5.8 [0.9–11.3] *p* = 0.12 3.7 [0.3–78.6] *p* = 0.29	reference 3.8 [2.0-7.8] ***p*** **< 0.001** 0.6 [0.2–1.7] *p* = 0.38		reference 0.4 [0.4–0.5] ***p*** **< 0.001** 0.6 [0.5–0.8] ***p*** **< 0.001**
School location						
urban (*n* = 1,302) semirural/rural (*n* = 1,050)	reference 2.0 [1.7–2.4] ***p*** **< 0.001**	reference 0.8 [0.6–1.1] *p* = 0.14	reference 1.7 [0.4–8.4] *p* = 0.51	reference 2.0 [1.1–3.7] ***p*** **= 0.02**		reference 0.6 [0.5–0.7] ***p*** **< 0.001**

*TTH* tension type headache, *pMOH* probable medication overuse headache, *H15+* headache on ≥ 15 days/month, *UdH* undifferentiated headache; significant p-values (< 0.05) are emboldened

**Table 6:** Adjusted analyses of associations (logistic regression) between headache types and demographic variables according to modified criteria for undifferentiated headache (mild-to-moderate headache lasting < 1 h)

Variable	Migraine	TTH	pMOH	Other H15+	UdH
	Adjusted odds ratio [95% confidence interval]; *p*				
Gender					
male female	reference 1.0 [0.8–1.2] *p* = 0.97	reference 0.9 [0.7–1.1] *p* = 0.25	reference 0.9 [0.2–4.1] *p* = 0.91	reference 1.8 [1.0-3.4] ***p*** **= 0.04**	reference 1.2 [1.0-1.4] *p* = 0.07
Age (years)					
6–11 12–17	reference 1.2 [1.0-1.4] *p* = 0.07	reference 1.9 [1.5–2.6] ***p*** **< 0.001**	reference 0.3 [0.0-1.5] *p* = 0.18	reference 2.8 [1.5–5.7] ***p*** **= 0.002**	reference 1.2 [1.0-1.4] *p* = 0.06
Altitude (metres above sea level)					
< 1,000 1,001–2,000 > 2,000	reference 1.4 [1.0-1.8] ***p*** **= 0.04** 1.2 [0.8–1.8] *p* = 0.29	reference 1.2 [0.8–1.8] *p* = 0.43 1.0 [0.6–1.9] *p* = 0.94	reference 8.0 [0.8–172.0] *p* = 0.09 8.6 [0.4–302.0] *p* = 0.18	reference 2.3 [0.8-6.0] *p* = 0.10 0.2 [0.0-0.8] ***p*** **= 0.04**	reference 0.5 [0.4–0.7] ***p*** **< 0.001** 0.7 [0.5–1.1] *p* = 0.12
Habitation					
urban semirural/rural	reference 1.7 [1.2–2.3] ***p*** **= 0.001**	reference 0.8 [0.5–1.3] *p* = 0.30	reference 0.5 [0.1–4.1] *p* = 0.48	reference 2.5 [1.1–6.8] *p* = 0.05	reference 0.8 [0.6–1.2] *p* = 0.28

*TTH* tension type headache, *pMOH* probable medication overuse headache, *H15+* headache on ≥15 days/month, *UdH* undifferentiated headache, significant *p*-values (<0.05) are emboldened

There were no associations between gender and migraine, TTH, pMOH or UdH, while other H15 + was more common among females than males according to logistic regression analysis (Table 6). TTH and other H15 + were significantly more common among adolescents in logistic regression analysis, migraine and UdH not quite significantly so (Table 6).

Migraine became increasingly prevalent at higher altitudes in bivariate analysis, while UdH was most prevalent at low altitude (Table 5). However, correction for other demographic variables weakened these associations above 2,000 m, with migraine and UdH showing inverse relationships with altitude (Table 6). Only migraine showed a clear association with habitation (more common in semirural or rural areas); other H15 + was more common in semirural or rural areas, and UdH less common, in bivariate analysis only (Tables 5 and 6).

### Headache yesterday

HY was reported by 488 participants (Table 7), who were 24.3% of those with headache in the preceding year and 20.7% of the total sample (this representing the observed 1-day prevalence). More females reported HY (29.6% of those with headache in the preceding year) than males (19.5%; *X*^*2*^(1, 2008) = 27.8; *p* < 0.001). No significant difference was seen between children (22.5%) and adolescents (25.5%; *X*^*2*^(1, 2008) = 2.3; *p* = 0.13). The age- and gender-adjusted 1-day prevalence was 19.9% (18.3–21.6).

**Table 7:** Reported and predicted headache yesterday, overall and by headache type according to modified criteria for undifferentiated headache (mild-to-moderate headache lasting < 1 h)

	Recalled mean headache frequency		Headache yesterday (proportion of those with headache [any, or of the specific type] in the preceding year)		
	Days in preceding 1 week (F7)	Days in preceding 4 weeks (F28)	Reported*n* (%)	Predicted (%)	
				from 1-week recall (F7*100/7)	from 4-week recall (F28*100/28)
Any headache (*n*=2,008)	1.2	2.9	488 (24.3)	17.6	10.3
males (*n*=1,060)	1.1	2.7	207 (19.5)	15.8	9.5
females (*n*=948)	1.4	3.1	281 (29.6)	19.7	11.2
children (*n*=804)	1.3	2.9	181 (22.5)	18.9	10.5
adolescents (*n*=1,204)	1.2	2.8	307 (25.5)	16.8	10.1
Migraine (*n*=940)	1.4	3.0	272 (28.9)	20.1	10.6
TTH (*n*=246)	1.0	2.3	51 (20.7)	15.0	8.1
pMOH (*n*=7)	4.1	21.7	5 (71.4)	59.2	77.6
Other H15+ (*n*=47)	4.4	17.9	37 (78.7)	62.9	64.0
UdH (*n*=755)	0.9	1.9	121 (16.0)	12.2	6.7

*TTH* tension type headache, *pMOH* probable medication overuse headache, *H15+* headache on ≥15 days/month, *UdH* undifferentiated headache

As expected, large proportions (> 70%) of those with H15+ (pMOH or other) reported HY (Table 7). Of the episodic headache types, migraine led with 28.9%, followed by TTH (20.7%) and UdH (16.0%).

For all headache types except pMOH, reported HY was higher than predicted from recalled headache frequency, whether the latter was based on the preceding 1 week or 4 weeks (Table 7). For all headache types except pMOH and other H15+, HY predicted from 1-week frequency was greater (up to 2-fold) than from 4-week frequency (Table 7).

## Discussion

This schools-based study in Nepal was the Global Campaign’s first in SEAR. We found that the vast majority of children and adolescents experienced headache in the preceding year: an estimated 83.9% corrected for age and gender, very similar to our earlier finding of 84.9% among adults in Nepal [8]). Migraine was the most common headache type. An estimated 2.2% had headache on ≥ 15 days/month, but only 0.3% in association with acute medication overuse (pMOH). Estimated 1-day prevalence was 19.9%.

The study highlighted the diagnostic difficulties commonly encountered in epidemiological studies among young people [3, 4, 8]. In large cross-sectional studies, headache diagnoses can be based only on subjective reporting of symptoms elicited by questionnaire. How questions are asked, and understood through the barriers of young age and linguistic and cultural diversities, are therefore pivotal. In previous papers [3–8] we have referred to children’s potential susceptibility to suggestion (built into leading questions), their tendency to favour affirmative responses, and their likely beliefs, reinforced in a school setting, that there are “right” and “wrong” answers. The diagnostic question set used in HARDSHIP, applying ICHD-3 criteria with regard to headache features and associated symptoms (criteria B-D), has not been formally validated: in these age groups, this is very difficult to do, requiring double interrogation with no benefit to the participant. It has, however, been used previously in nine languages and six countries [3–8], always translated in accordance with the Global Campaign’s translation protocol [16], with subsequent assessment of comprehensibility in a sample of the population of interest. In addition, in these previous studies, self-completion of questionnaires has been mediated, as it was here, by the class teacher or investigator to assist comprehension when needed. These measures have not entirely obviated diagnostic uncertainties. In particular, in countries in sub-Saharan Africa, very high proportions reporting symptoms usually associated with migraine have led to unfeasibly high estimates of migraine prevalence (definite + probable) [3, 4, 8]. Furthermore, specifically in Nepal, our previous study among adults encountered almost universal reporting of photophobia by those with headache, so that this symptom had no value in differential diagnosis [9]. In the present study, almost 95% of those with headache reported phonophobia (94.6%), and an unlikely proportion reported vomiting (43.0%). Also troublesome was that the conventional diagnostic criteria for UdH (mild headache lasting < 1 h) did not behave as in most previous studies [4–8], capturing only 4.8% of participants because very few reported mild headache (7.8%).

Our solution here was to modify the criteria for UdH, to include moderate headache but retaining the essential criterion of short duration (< 1 h). In justification, distinction between mild and moderate headache (the actual terms used were “not bad” [in Nepali: garho hudaina] and “quite bad” [madhyam garho]) is obviously highly subjective at any age, and diagnostic distinction ought not to turn upon this. The result of this modification was prevalence estimates for UdH of 31.7% (up from 4.8%) and for migraine of 39.8% (still high, but down from a very improbable 58.4%).

The observed 1-year prevalence of any headache (85.4%) falls near the higher end of the range (61.3–88.5%) found in other schools-based studies in this series conducted by the Global Campaign [3–8]. Higher prevalence among females than males, and among adolescents than children, are universal in these studies [3–8]. Both in Benin [3] and Zambia [8], estimates of migraine prevalence were implausibly high (53.2% and 53.4% respectively), with diagnosis driven by high proportions reporting nausea and/or vomiting [8] or photophobia and phonophobia [3]. In both countries, substantial proportions of those classified as probable migraine reported usual headache durations of < 1 h [3, 8]. In these studies, modification of the criteria for UdH to include moderate headache would markedly reduce the estimates of migraine prevalence, bringing them into the plausible range. We suggest this supports this modification.

With the modified criteria applied, observed prevalence of headache overall was higher among adolescents than among children (77.3% vs. 91.8% [Table 4]), as were estimates for migraine, TTH, UdH and other H15+, although increases in migraine and UdH did not quite reach significance after adjustment (Table 6). As a proportion of all headache, UdH declined very slightly among adolescents. However, these differences were small: headache is highly prevalent even among children in Nepal, at levels comparable to those among adults [9].

On this last point, the present study is nicely complementary to our previous study in adults [9, 10], informing health (and educational) policy in Nepal that headache affects the great majority of those aged 6–17 years as well as the great majority of adults aged 18–65. There are differences, of course: principally in the occurrence in children and adolescents of UdH (believed to be headache in the developing brain yet to manifest as migraine or TTH [6]), but there is also a strikingly lower prevalence of H15 + among children and adolescents (2.2% vs. 7.4% among adults). MOH is known to take time to develop [20]. Other H15 + was likely to have included chronic migraine and chronic TTH, which evolve from their episodic precursors, again over time [21, 22]. This highlights the theoretical opportunity for preventative intervention in childhood and adolescence to avert much lost health in adulthood – an attractive postulate, but unfortunately it remains unsupported by empirical evidence.

An important concordance between the present results and those of our adult study in Nepal is the positive association between migraine and altitude, appearing in both studies to weaken above 2,000 m [23]. In a recent similar adult study in Peru, we found a more robust association [24]. This, therefore, is the third time we have been able to demonstrate this relationship, and, to our knowledge, the first time it has been demonstrated in children and adolescents. The cause remains unclear [23]. Our finding here that UdH was negatively associated with altitude, in a manner that was the inverse of that of migraine, is thought-provoking: what would manifest as UdH at low altitude perhaps acquires the attributes of migraine at high altitude.

Finally, we note that the 20.7% of the sample reporting HY (24.3% of those reporting any headache in the preceding year) were a clearly higher proportion than predicted from reported headache frequency, which was based on recall, with 1-week recall (17.6%) giving a closer match than 4-week recall (10.3%). This pattern was repeated across genders and ages for all the episodic headaches (Table 7). On this evidence, children and adolescents underreport headache frequency – as we have seen in previous schools-based studies [3–8]. In fact, this tendency has been a robust finding across geography, culture and age, since it has been reported in adult studies also [24–27] – though to a lesser degree, perhaps because adults have a better sense of time.

Because, in Global Campaign methodology, UdH takes diagnostic precedence over migraine and TTH [7], prevalence estimates from studies using this methodology cannot sensibly be compared with those that have not taken account of UdH. Many published studies of children and adolescents have found large proportions of unclassified headache that might have been UdH but were not identified as such (for discussion of this, see [7]).

The strengths of this study were in the use of standardised methodology, albeit with modifications to the questionnaire [14, 15], coupled with an adequate sample size derived from almost nationwide sampling. As noted in Methods, we could not sample in Province 7. Most characteristics of Province 7 (cultural, geographic and economic) are a mixture of those of Provinces 5 and 6, all remote and relatively underdeveloped. These three provinces have similar literacy levels, life expectancies, ethnic diversity, traditional lifestyle and religious practices [28, 29]. The principal limitations were those inherent in cross-sectional surveys, and in the uncertain reliability of information gathered from children. The additional uncertainties in recall over the past 4 weeks were countered by separate enquiry into HY. Already discussed in length are the diagnostic issues, and our modification of the standard HARDSHIP algorithm with regard to UdH. There may be an underlying limitation in the ICHD criteria themselves in that they do not apply well to children, particularly with regard to duration [30]. We believe this underpins the importance of including UdH as a separate diagnosis, rather than stretching the criteria for migraine as has been suggested [30], although it is not yet clearly established how UdH should be defined.

## Conclusion

These findings complement those of our previous study in adults, showing that headache is highly prevalent also among children and adolescents in Nepal, and confirming the positive association between migraine prevalence and altitude of dwelling. The finding of less pMOH and other H15 + among children and adolescents than among adults suggests an opportunity to avert lost health in adulthood through preventative intervention in childhood and adolescence.

A separate paper will describe headache-attributed burden, including functional impairment and lost time from school and other activities, which will be more informative to health and educational policies.

## Data Availability

The original data are held at Tribhuvan University Teaching Hospital, Kathmandu, Nepal, and the analytical set at University of Mersin, Mersin, Turkey, and at Norwegian University of Science and Technology, Trondheim, Norway. When analyses are completed, anonymised data will be available on request for academic purposes, in line with the policy of the Global Campaign against Headache.

## Abbreviations

AOR: Adjusted odds ratio
CI: Confidence interval
d/m: days/month
GBD: Global Burden of Disease (study)
H15+: Headache on ≥15 days/month
HARDSHIP: Headache-Attributed Restriction, Disability, Social Handicap and Impaired Participation (questionnaire)
HY: Headache yesterday
ICHD: International Classification of Headache Disorders
LTB: *Lifting The Burden*
MOH: Medication-overuse headache
NorHead: Norwegian Centre for Headache Research
pMOH: Probable MOH
SD: Standard deviation
SEAR: South East Asia Region
TTH: Tension-type headache
UdH: Undifferentiated headache
